# Oxygen Consumption Rate Analysis of Mitochondrial Dysfunction Caused by *Bacillus cereus* Cereulide in Caco-2 and HepG2 Cells

**DOI:** 10.3390/toxins10070266

**Published:** 2018-07-02

**Authors:** Marlies Decleer, Jelena Jovanovic, Anita Vakula, Bozidar Udovicki, Rock-Seth E. K. Agoua, Annemieke Madder, Sarah De Saeger, Andreja Rajkovic

**Affiliations:** 1 Department of Food Technology, Food Safety and Health, Faculty of Bioscience Engineering, Ghent University, Coupure Links 653, 9000 Ghent, Belgium; mdecleer@hotmail.com (M.D.); Jelena.Jovanovic@UGent.be (J.J.); rockseth@gmx.fr (R.-S.E.K.A.); 2 Laboratory of Food Analysis, Department of Bioanalysis, Faculty of Pharmaceutical Sciences, Ghent University, Ottergemsesteenweg 460, 9000 Ghent, Belgium; Sarah.DeSaeger@UGent.be; 3 Department of Food Preservation Engineering, Faculty of Technology, University of Novi Sad, Bulevar Cara Lazara 1, 21000 Novi Sad, Serbia; anita.vakula@hotmail.com; 4 Department of Food Safety and Food Quality Management, Faculty of Agriculture, University of Belgrade, Nemanjina 6, 11081 Zemun-Belgrade, Serbia; bozidar.udovicki@agrif.bg.ac.rs; 5 Department of Organic and Macromolecular Chemistry, Organic and Biomimetic Chemistry Research Group, Faculty of Sciences, Campus Sterre, Krijgslaan 281, Building S4, 9000 Gent, Belgium; Annemieke.Madder@UGent.be

**Keywords:** cereulide, emetic toxin, *Bacillus cereus*, Seahorse XF, extracellular flux, mitochondrial dysfunction, respiration, oxygen consumption rate, depsipeptides

## Abstract

The emetic syndrome of *Bacillus cereus* is a food intoxication caused by cereulide (CER) and manifested by emesis, nausea and in most severe cases with liver failure. While acute effects have been studied in the aftermath of food intoxication, an exposure to low doses of cereulide might cause unnoticed damages to the intestines and liver. The toxicity which relies on the mitochondrial dysfunction was assessed on Caco-2 and HepG2 cells after exposure of one, three and ten days to a range of low doses of cereulide. Oxygen consumption rate analyses were used to study the impact of low doses of CER on the bioenergetics functions of undifferentiated Caco-2 and HepG2 cells using Seahorse XF extracellular flux analyzer. Both Caco-2 and HepG2 cells experienced measurable mitochondrial impairment after prolonged exposure of 10 days to 0.25 nM of cereulide. Observed mitochondrial dysfunction was greatly reflected in reduction of maximal cell respiration. At 0.50 nM CER, mitochondrial respiration was almost completely shut down, especially in HepG2 cells. These results corresponded with a severe reduction in the amount of cells and an altered morphology, observed by microscopic examination of the cells. Accurate and robust quantification of basal respiration, ATP production, proton leak, maximal respiration, spare respiratory capacity, and non-mitochondrial respiration allowed better understanding of the effects of cereulide in underlying respiratory malfunctions in low-dose exposure.

## 1. Introduction

Cereulide (CER) is a toxin notorious for being the causative agent of an acute *Bacillus cereus* emetic food intoxication [1,2,3]. This emetic syndrome, characterized by nausea, vomiting and malaise 0.5–5 h after ingestion of food containing cereulide, is usually mild and self-limiting. However, in some cases the food poisoning leads to severe clinical pathologies including liver failure with rhabdomyolysis [4], or liver failure with acute encephalopathy and a dysfunction of the beta-oxidation process [5]. CER is a cyclic and lipophilic dodecadepsipeptide (1.2 kDa) that acts as a potassium ionophore on the mitochondrial membrane and is structurally related to a known antibiotic valinomycin [6]. Both cereulide and valinomycin are known K^+^ ion-selective ionophores. Cereulide and valinomycin have 12 stereogenic centers containing very similar sequence of cyclo [-d-O-Leu-d-Ala-l-O-Ala-l-Val-]_3_ in cereulide and cyclo [-d-O-Val-d-Val-l-O-Ala-l-Val-]_3_ in valinomycin. As K^+^ ion-selective ionophores both cereulide and valinomycin cause a potassium-dependent drop in the transmembrane potential of mitochondria. As such, both compounds may affect mitochondrial function. Some differences in their biological activity may be explained by differences in their chemical properties, including differences in amino acid composition. Cereulide is reported to exibit the K+-ion-selective ionophore property at a lower concentration than valinomycin [7]. 

As with valinomycin [8,9,10,11], cereulide is produced through a unique nonribosomal peptide biosynthesis [12,13]. It is formed in food during late exponential and stationary growth phases of *B. cereus* growth [14] and is highly resistant towards heat (80 min at 121 °C and 60 min at 150 °C at pH 9.5; no inactivation at 121 °C and 150 °C at pH of 9 and below [15]), pH (pH range from 2 to 11), and proteolytic enzymes such as pepsin and trypsin [15,16]. As a consequence, food processing and preparation or reheating of cooked foods prior to consumption will not destroy CER, and the intact toxin will pass the stomach and reach the intestines without the loss of its activity. Most of the reported cases were related to food leftovers or takeaway pasta and rice dishes that were improperly stored, allowing the growth of *B. cereus* with production of CER [1,17]. Reported studies generally focused on starch-rich foods (mainly rice and pasta) linked with foodborne outbreaks, while the prevalence of CER in other food categories is somewhat less described [18]. The acute intoxication dose provoking clinical emetic manifestations was estimated at ca. 8–10 μg CER per kg of body weight [19,20,21]. Prevalence data on CER in food samples not related to foodborne outbreaks are less reported and generally indicate low CER concentration in tested samples [22,23]. A Belgian study conducted on rice dishes collected in Chinese-style restaurants revealed that CER was found in 7.4% of the samples, with an average CER concentration of 4 µg·kg^−1^ food [17]. This concentration is much lower compared to the levels found in foods incriminated in foodborne outbreaks, such as for example 1 μg/g to 10 μg/g as reported for an outbreak in a kindergarten in Norway [24]. Besides the acute effects associated with food poisoning, a repeated exposure to sub-emetic doses (doses that do not cause visible emetic symptoms and thus result in an un-noticed exposure) of CER present in food from restaurants or households that was improperly stored might be potently harmful [25,26,27].

Although *B. cereus* is ubiquitously distributed in the environment, it is known that only emetic strains of *B. cereus* that possess the *ces* gene have the potential to produce CER [28]. Messelhausser et al. investigated the prevalence of emetic strains in foods of different origin (*n* = 3654) and reported that about 1% of tested samples contain the emetic strain [29]. However, the emetic strains were not only detected in the expected farinaceous foods but also in vegetables, fruit products, sauces, soups, and salads and in cheese and meat products. Although the prevalence was very low, a relatively higher incidence of emetic *B. cereus* strains was found in tested ice cream samples (4.7%) [30]. A study conducted on different foods on the Dutch market revealed that even 16.8% of the tested isolates produced CER and/or contained the *ces* gene [23]. In clinical stool samples analyzed in South Korea, 20% of *B. cereus* isolates harbored *ces* genes in comparison to ca. 34–87% harboring (some of) enterotoxins genes. Relatively lower prevalence of *cer* + *B. cereus* and the fact that CER is not produced at refrigeration temperatures (t_min_ for CER is considered to be 10–12 °C) contribute to the relatively low prevalence of CER in food. In addition, amounts of CER produced at 12° (are much smaller than at 30–37 °C (2–8 μg/g in bacterial wet weight and 31–194 μg/g bacterial wet weight, respectively) [31].

Since CER causes gastroenteritis symptoms and the human exposure is predominantly via food, the gut is the first place in the human body exposed to the toxin. In view of this, it is of great importance to investigate the effects of various food-relevant CER concentrations on the models of intestinal epithelium. To mimic the contact of CER in food with the human digestive tract in the current study the Caco-2 cell line (human colorectal adenocarcinoma) was selected as model for the intestinal epithelial barrier. In addition, expected hepatoxic effects were assessed using HepG2 cells as a model for human hepatocytes. Considering known mitochondrial toxicity of CER, the mitochondrial respiration of Caco-2 and HepG2 was characterized as an indicator of cellular metabolism and fitness in the function of the CER exposure by extracellular flux analysis of oxygen consumption rate (OCR). Measurements of OCR deciphered key parameters of mitochondrial respiration when different aspects of the electron transport chain were shut down. Through the use of mitochondrial inhibitors, six mitochondrial respiration parameters can be determined: basal, ATP production-linked, maximal, proton leak-linked OCR, spare respiratory capacity and non-mitochondrial respiration [32,33]. For this purpose, the Agilent Seahorse XF24 technology was used to evaluate mitochondrial dysfunction in the absence of cell death caused by (long-term) exposure of Caco-2 and HepG2 cells to low sub-emetic concentrations of CER.

## 2. Results

### 2.1. Determination of Subacute Concentrations of CER on Caco-2 and HepG2 Cells

**Figure 1:**
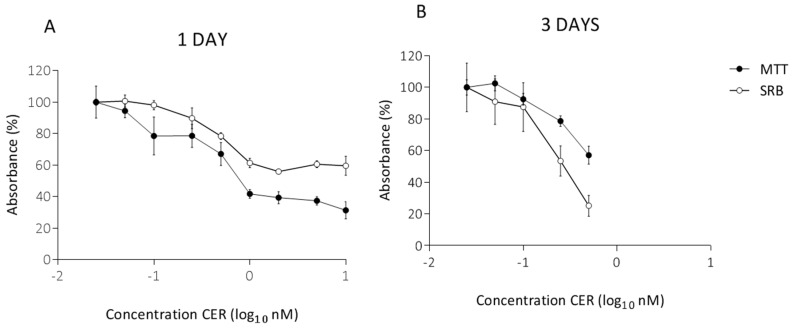
MTT assay and SRB assays after 1 day (**A**) and 3 days (**B**) treatment with cereulide (CER) in undifferentiated Caco-2 cells. The absorbance is expressed as a percentage of the control values (0 nM CER). Data (*n* = 12, four technical replicates and three independent repetitions) are presented as mean values with standard deviations.

In preliminary experiments, the sub-acute concentrations were determined by exposing Caco-2 cells to a range of CER concentrations for 24 h and 72 h. After 1 day, the results of the MTT assay indicated a significant decrease (*p* < 0.05) in cell viability, with a decrease of more than 50% starting at a concentration of 1 nM CER. A similar, visually less pronounced decrease was observed in the SRB assay (Figure 1A). Hence, higher concentrations, which already gave a significant decrease in cell viability after 1 day, were omitted for the 3-day exposure (Figure 1B). According to the MTT assay, three days of CER treatment significantly decreased Caco-2 cell viability with 21% and 43% compared to the untreated cells after exposure to 0.25 and 0.50 nM CER, respectively (Figure 1B). CER induced significant reduction (*p* < 0.05) in protein content in the SRB assay at ≥0.25 nM (Figure 1B). The selected treatments (0.05–0.50 nM) induced toxicity, but the majority of the cells survived the exposure. Based on these findings, the same concentration range was used for the subsequent study of long-term, 10-day, exposure. 

**Figure 2:**
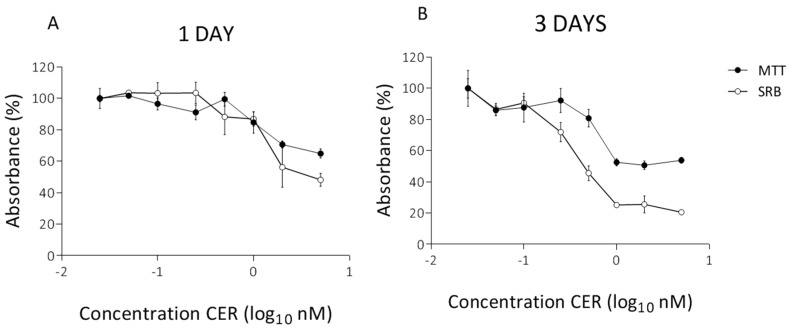
MTT assay and SRB assay after 1 day (**A**) and 3 days (**B**) treatment with CER in undifferentiated HepG2 cells. The absorbance is expressed as a percentage of the control values (0 nM CER). Data (*n* = 12, four technical replicates and three independent repetitions) are presented as mean values with standard deviations.

In parallel, similar experiments were performed on undifferentiated HepG2 cells. As shown in Figure 2A, CER concentrations of 2 nM and 5 nM resulted in a decrease of 30% and 35% of cell viability after 24 h measured by the MTT assay. The toxicity was more pronounced based on the SRB results, with reductions of 44% for 2 nM and 52% for 5 nM (Figure 2A). However, at these concentrations cells continued to grow, and thus the same concentrations were used for an exposure of 3 days. Clearly, the three-day treatment significantly reduced the cell viability compared to the one-day treatment (Figure 2B). At 1 nM of CER and higher, viability of HepG2 was severely compromised deceasing to about 50% as indicated by the MTT assay. The decrease in SRB absorption measurement were even more drastic with a reduction of about 75%, indicating the impact on both metabolic activity of cytoplasmic enzymes and cells and total protein amount. At these concentrations (≥1 nM) present cells continued to function, with CER induced cellular alternations. To ensure sufficient number of living cells after 10 days of exposure, the maximum exposure concentration was reduced to 0.50 nM CER for the subsequent experiments.

### 2.2. Long-Term Effects of Cereulide on Mitochondrial Respiration of Caco-2 and HepG2 Cells

**Figure 3:**
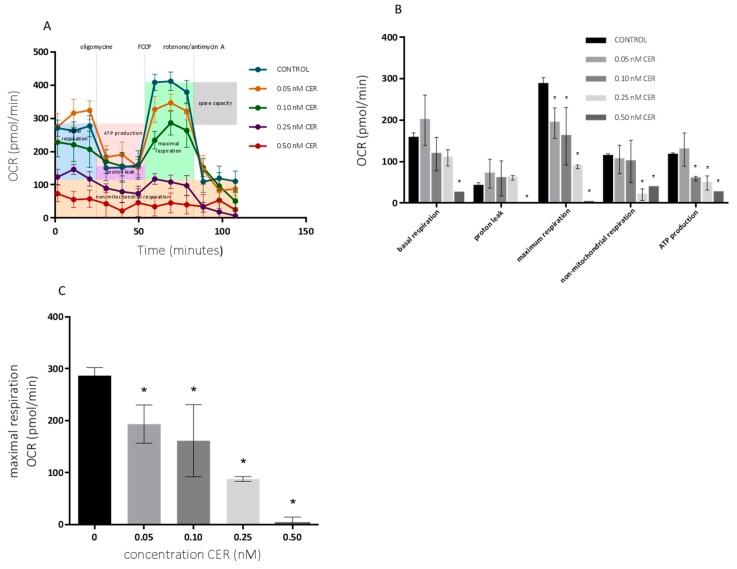
Effects of 10-day cereulide treatments on mitochondrial respiration of Caco-2 cells. (**A**) Mitochondrial profile; (**B**) mitochondrial parameters; (**C**) maximal respiration. Data (*n* = 12, four technical replicates and three independent repetitions) are expressed as mean ± SD. * indicated a significant difference at *p* < 0.05.

**Figure 4:**
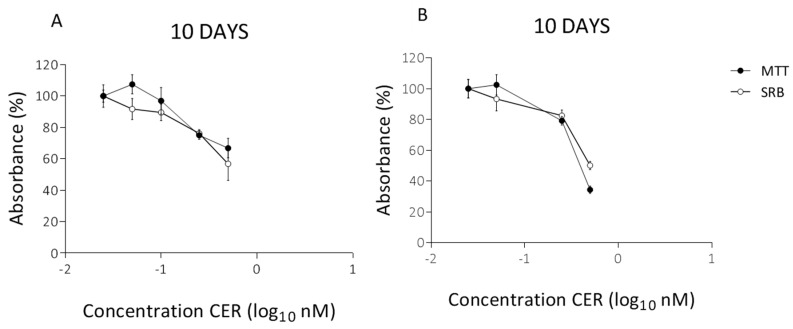
MTT assay and SRB assay at the end of the treatment (10 days) with CER in undifferentiated Caco-2 cells (**A**) and HepG2 cells (**B**). The absorbance is expressed as a percentage of the control values (0 nM CER). Data (*n* = 12, four technical replicates and three independent repetitions) are presented as mean values with standard deviations.

**Figure 5:**
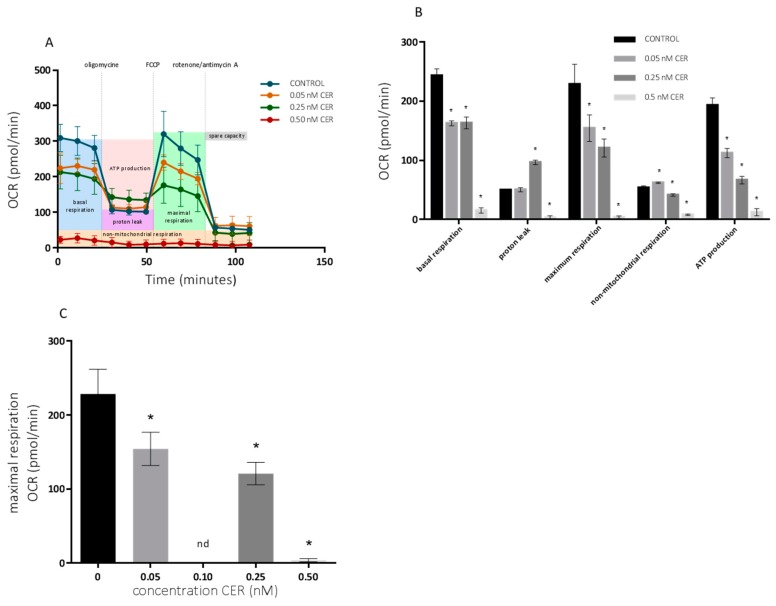
Effects of 10-day cereulide treatments on mitochondrial respiration of HepG2 cells. (**A**) Mitochondrial profile; (**B**) mitochondrial parameters; (**C**) maximal respiration. Data (*n* = 12, four technical replicates and three independent repetitions) are expressed as mean ± SD. * indicated a significant difference at *p* < 0.05.

The effects on the mitochondrial respiration after 10-day exposure with varying doses of CER were determined using the Seahorse XF Cell Mito Stress Test kit. The basal respiration and respiration after sequential injection of oligomycin, Carbonyl cyanide-4-(trifluoromethoxy) phenylhydrazone (FCCP) and rotenone + antimycin A are depicted in Figure 3 for undifferentiated Caco-2 cells. These results are accompanied with MTT and SRB profiles shown in Figure 4. The OCR profile for HepG2 cells, is depicted in Figure 5. 

As shown in Figure 3, the mitochondrial respiration of the undifferentiated Caco-2 cells was compromised starting at 0.25 nM CER. The toxic effects of CER were reflected in a decreased non-mitochondrial respiration, ATP-linked respiration and especially in maximal respiration. Maximal respiratory capacity was estimated by an FCCP-stimulated respiration and observed decrease is a strong indicator of potential mitochondrial dysfunction [34]. Treatments with 0.05 nM CER had small effects on respiration, with the exception of a significant reduction in maximum respiration, which could not be observed with MTT, and SRB assays (Figure 4A). Treatments with up to 0.10 nM CER did not affect the basal respiration of the Caco-2 cells, but produced a significant reduction (*p* < 0.05) in the maximum respiration and ATP turnover. Higher concentrations of CER (0.25 and 0.50 nM) decreased mitochondrial respiration of cells even at the basal state, and their metabolic activity has been largely decreased so that no effects of modulators were observed. Note that the MTT/SRB results did not indicate significant reduction after treatment with 0.10 nM CER and even for higher concentrations only moderate toxicity was observed (Figure 4A). 

Figure 5 depicts the OCR pattern of undifferentiated HepG2-cells exposed to CER (0.05–0.50 nM).

Similar effects have been seen with a significant reduction (*p* < 0.05) of ATP-linked respiration and maximal respiration starting at 0.05 nM CER. After exposure to 0.50 nM CER, all mitochondrial functions of the cells were severely compromised, whereas in Caco-2 cells comparable effects started at 0.25 nM CER. Maximal respiration significantly decreased with 50% following 0.25 nM CER treatment, while a 98% decrease was observed after 0.50 nM CER exposure compared with the control. In parallel, the mitochondrial ATP production declined to 58% (0.05 nM CER), 34% (0.25 nM CER) and 6% (0.50 nM) of that of the untreated group. The experiments conducted in both cell lines indicated that various CER treatments caused perturbations in the mitochondrial respiration, although the effects were more pronounced in HepG2 cells. In both cell lines, the maximal respiration decreased in a dose-dependent manner (Figure 3 and Figure 5). 

The generation of ATP through oxidative phosphorylation is one of the predominant physiological functions of mitochondria. Since CER acts on mitochondria by uncoupling of oxidative phosphorylation, the results confirmed the expected decrease in ATP production with increasing CER concentration. The uncoupling effect of cereulide on mitochondrial respiration was previously reported to be similar to those of uncouplers 2,4-dinitrophenol (DNP), carbonylcyanide m-chlorophenylhydrazone (CCCP), and valinomycin [35]. The same authors reported the minimum cereulide concentration to detect uncoupled oxygen consumption was 50 ng·mL^−1^ measured by oxygen reduction in the reaction medium with isolated rat mitochondria. In our study, both maximal respiration and ATP-linked respiration proved to be sensitive parameters for determination of the mitochondrial dysfunction especially when compared to MTT and SRB assays. As a consequence, also cell spare respiratory capacity (SRC) was impaired indicating an affected ability of the exposed cells to cope with sudden increased need for ATP. The mitochondrial SRC is regarded as an important aspect of the mitochondrial function and is given by the difference between maximal and basal cellular OCR. When cells are subjected to stress, energy demand increases, with more ATP required to maintain cellular functions. A cell with a larger spare respiratory capacity can produce more ATP and overcome more stress, which indicates that this is an estimative of the cell’s ability to cope with large increases in ATP turnover. Consequently, CER exposure, which is negatively affecting the mitochondrial function, possibly exerts negative effects on the ability of cells to cope with other stresses. This is in agreement with expected impact of CER on mitochondria by depolarization of the mitochondrial inner membrane and impairment of ATP synthesis [36] by facilitating the passage of K+ ions [6,35]. The impact of CER on mitochondrial ATP synthase that also translocates protons across the inner membrane has been best observed after the addition of oligomycin. The presence of oligomycin during the estimation of maximal respiration is important to prevent the reverse activity of ATP synthase with rapid intracellular ATP depletion, which may lead to cellular metabolic dysfunction and death. Hence, recent data showed that the presence of oligomycin significantly underestimates FCCP-induced maximal OCR and SRC [37]. Finally, the addition of a potent respiratory chain inhibitor, such as antimycin A, allows the estimation of non-mitochondrial OCR. The mitochondrial respiratory chain in the inner membrane is proton translocating; H^+^ are pumped out of the matrix space when electrons are transported along the chain. The considerable impact of CER on the metabolic fate of exposed cells is based on the fact that mitochondria carry out most cellular oxidations and produce the bulk of the cell’s ATP. Large amounts of NADH (and FADH_2_) are produced by the oxidation reactions. The energy available from combining oxygen with the reactive electrons carried by NADH and FADH_2_ is harnessed by the electron-transport chain. This respiratory chain pumps H^+^ out of the matrix to create a transmembrane electrochemical proton gradient. In turn, the transmembrane gradient is used both to synthesize ATP and to drive the active transport of selected metabolites across the mitochondrial inner membrane [38]. 

Additionally, the Seahorse XF mitochondrial stress test was confirmed by MTT and SRB assays (Figure 4B). Hence, the reduction in cell viability was rather low compared to the findings of the Seahorse XF OCR measurements. In both cell lines, cell viability started to decrease significantly at 0.25 nM CER with a small reduction around 25% and 20% for Caco-2 cells and HepG2-cells, respectively. Especially for the highest concentration (0.50 nM CER), the effects were more pronounced according to maximal OCR. Exposure to 0.50 nM resulted in a critically compromised OCR profile, while, based on the SRB assay, 56% and 50% of the Caco-2 and HepG2cells respectively were still viable. The findings indicated that the MTT/SRB assays proved to be less sensitive to the certain cytotoxic effects of CER. Or in other words partial inhibition of the OCR does not have measurable effect on cell density in Caco-2 and HepG2 cells. Moreover, measurements of fluxes give more information about the ability of mitochondria to make ATP than do measurements of membrane potential that is diminished by cereulide [25,39]. For the experiments with intact cells as done in the current study, the best assay is the equivalent measurement of cell respiratory control, which reports the rate of ATP production, the proton leak rate, the coupling efficiency, the maximum respiratory rate, the respiratory control ratio and the spare respiratory capacity. This information is important to establish whether the mitochondria in a stressed cell still generate ATP or are damaged and maintain their membrane potential by hydrolysing glycolytic ATP. 

Other in vitro toxicity studies indicated that, at extremely low doses, CER was toxic towards porcine fetal Langerhans islets and beta cells. Within 2 days, 1 ng·mL^−1^ of CER caused necrotic cell death of the islet cells hampering their insulin content [40]. In whole mouse islets apoptotic cell death was observed in 75% of the islets after 72 h exposure with 0.25 ng·mL^−1^ CER [25]. These results indicated that beta cells are even more sensitive to CER exposure than HepG2 cells. Nonetheless, similar to our study, very low doses of CER impaired the mitochondrial activity of the cells. In the case of beta cells this leads to impaired insulin secretion and cell death which both play key roles in the regulation of the diabetes [41].

The exposure to CER slowed down the cell growth starting from a concentration of 0.10 nM CER. Standard examination of the microscopic pictures revealed vacuolization in cells at 0.10 nM CER and higher. In parallel, the reduction in basal respiration observed using the Seahorse XF Technology corresponded to the decrease in cell number. Exposure with 0.50 nM CER induced cytotoxicity as the cells were reduced in number as well as in size. Hence, based on the results of MTT and SRB assays, the remaining cells appeared to be alive and active. In contrast, at this concentration mitochondrial respiration could barely be observed in the Seahorse XF Cell Mito Stress Test. This suggested that the microscopic observations corresponded to the data found with Seahorse XF Cell Mito Stress Test. At the end of the HepG2 treatment period with 0.50 nM CER, the cell morphology was severely impaired. The number and size of cells was highly reduced and the remaining cells were barely viable as indicated by the MTT and Seahorse experiments. The results revealed that HepG2 cells were relatively more sensitive to the measured cytotoxic effects of CER compared to Caco-2 cells. Considering the dilution of cereulide in human body after the ingestion, the amounts of CER reaching target cells are not known at the moment. Future physiologically-based kinetic (PBK) models are to give insight into dose-, species-, matrix-and interindividual human variation-dependent effects of cereulide. It is however clear that cereulide at very low doses induces damages in diverse organs and cells, and that doses used in this study complement previous studies. They also add value to the hypothesis that cereulide can causes cell death at low doses, and cell dysfunction at even lower doses, not only in here tested HepG2 and Caco-2 cells. The current findings further depict relevance of these low doses in mitochondrial respiration impairment that can result cell dysfunction. Recently, a decreased basal respiration was noted in beta cells following 24 h exposure to 0.25–0.5 ng·mL^−1^ CER [25,41]. Based on current findings we may assume that other aspects than basal respiration may be even more influences by the observed mitochondrial damages. Other potential pathological traits, in addition to the observed mitochondrial impairment, should be considered at these doses, as well. Cereulide is most likely not specific in its channel forming in biological membranes and acting upon cross cell-membrane gradients of potassium ions can impair different metabolic regulators in diverse mammalian cells, for example, the cells of innate and acquired immunity or voltage-gated potassium channel (hERG) heart muscle cells [36]. Testing different cells types and assessing potential risks for functional impairments is among other factors supported by reported findings that show that CER was found in the heart, brain, muscle and fat tissue of the piglets fed with feed containing CER [42]. While the exact ratios of external and internal doses are not yet clear, and the investigations are also influenced by the sensitivity of analytical methods used, one still needs to test effects of lowest detectable concentrations. The absolute values and even the ratios between different aspects of mitochondrial respiration following the exposure to low doses of CER may vary depending on the medium in which the respiration analysis are done, and this should be taken into account when extrapolating current findings to other experimental conditions [43]. 

It is also important to note that current experiments used established cell cultures that only partially mimic actual physiological situations in humans. In the case of well-established Caco-2 cells it is known that they express a large number of enzymes and transporter proteins present in normal human intestinal epithelium, but it is also known that the gene expression profiles of transformed epithelial cell lines like Caco-2, HT29 and normal human intestinal epithelium differ from each other. However, the differences are not only found between the cell lines and normal epithelium, but also between different cells lines, such as Caco-2 and HT29 cell lines [44,45,46,47]. Normal intestinal epithelium is made up of several different cell types, and differences in gene expression profiles are not only observed in the mucosal epithelium along the whole gastrointestinal tract, but also along the crypt-villus axis [47]. Therefore, one should not directly extrapolate current or any other in vitro findings to in vivo systems. Still, intestinal epithelial cell models such as the Caco-2 cell model hold many advantages due to their simplicity and reproducibility allowing inter-laboratory comparison of results. Furthermore, pursuing an effect toxin in a cell line model opens the possibility of studies of molecular mechanisms which may be more difficult to address in vivo [48]. Also, for HepG2 cells, many positive sides and some downsides can be noted in comparison to primary human hepatocytes, which are considered as the gold standard model for cytotoxicity studies. However, the scarce availability of fresh human liver samples, complicated isolation procedures, limited life span, inter-individual variability, as well as significant costs pose a profound obstacle in the use of primary hepatocytes [49,50]. Therefore, immortalized liver-derived cell lines were proposed as an alternative for their unlimited availability and phenotypic stability. A first alternative is the widely used human hepatocellular carcinoma cell line HepG2. These cells are highly differentiated and display many of the genotypic features of normal liver cells. Even their main limitation that is linked to their low metabolic capacities compared with primary hepatocytes [51] and which makes them appropriate for testing the toxicity of the parent molecule but less suited for metabolite toxicity testing was not an obstacle in our study. The latter is due to the hypothesis that CER due its chemical stability does not metabolize in the liver, which is proven by analysis of different tissues and body fluids in patients suffering CER intoxication [5,52].

## 3. Conclusions

Mitochondrial damage has been proposed as the mechanism underlying the induced hepatotoxicity and intestinal cell damage associated with CER intoxication. Our in vitro studies have shown that both Caco-2 and HepG2 cells are highly susceptible for the toxic effect of very low CER concentrations. The concentrations tested are lower than those found in foods on the market or even those reported in body fluids of patients diagnosed with severe forms of CER intoxication, such as gastric fluid (4 ng·mL^−1^), blood serum (4 ng·mL^−1^), and urine (8 ng·mL^−1^) and stool samples (160–800 ng/g) [52]. A long-term exposure of 10 days with 0.25 nM CER seemed to be a critical concentration in both cell lines. However, the effects were even more pronounced in HepG2 cells. This might suggest that chronic low exposure of CER might exert damaging effects at liver cell level. As indicated in this in vitro study, CER exerts harmful effects to liver and epithelial colon cells at very low doses, which are not associated with visual symptoms such as vomiting. The literature indicated that low doses of CER are found in foods with no food intoxication history, supporting the hypothesis of possible hidden exposure [17].

The use of Seahorse XF extracellular flux analysis of mitochondrial respiration is a suitable model for the identification of mitochondrial toxicity of CER. When no significant reduction in cell viability was observed based on MTT and SRB results, the Seahorse XF Technology was able to detect a reduced maximal respiration, which suggested that toxic impact might be underestimated or go unnoticed based on MTT/SRB alone. This also confirms the importance of the role of mitochondrial dysfunction in the absence of cell death when studying low doses of toxins [53]. Moreover, the use of respiration modulators in Seahorse XF analysis provided critical information about the key parameters of mitochondrial functions, which could not be evident in the measurements of basal respiration alone. Especially the maximal OCR appeared to be more sensitive to the presence of CER than other currently used assays for CER detection such as in vitro boar semen bio-assay where the threshold concentration provoking visible mitochondrial damage was 2–4 ng·mL^−1^ [18,19,54]. Taken together, chronic or repeated exposure to low doses of CER that are too low to cause visible effects of emesis is a potential threat for human health. 

## 4. Materials and Methods

### 4.1. Materials and Reagents

Dulbecco’s modified Eagle’s medium (DMEM), GlutaMAX™, penicillin/streptomycin and non-essential amino acids were purchased at Life Technologies (Merelbeke, Belgium), whereas foetal bovine serum was from Greiner Bio-One (Vilvoorde, Belgium). Phosphate buffered saline (PBS) without Ca^2+^, Mg^2+^, was obtained from Westburg (Leusden, The Netherlands) and trypsin-EDTA 0.05% from Thermo Fisher Scientific (Merelbeke, Belgium).

### 4.2. Cell Culture

Caco-2 (human colorectal adenocarcinoma) and HepG2 (human hepatocellular carcinoma) cells were obtained from ATCC and cultivated in Dulbecco’s Modified Eagle’s Medium (DMEM) containing GlutaMAX™, 4.5 g/L d-glucose and pyruvate supplemented with 10% fetal bovine serum, 1% non-essential amino acids and 1% penicillin/streptomycin. The passage number of the cells used in this study was maintained between 12 and 25. Cells were grown in T25 (25 cm^2^) polystyrene cell culture flasks in a humidified chamber with 5% CO_2_ at 37 °C and 95% air atmosphere at constant humidity. The culture medium of the cells was changed every other day and cell morphology was daily checked by visual inspection with phase-contrast microscopy (Leica DMIC, Leica Microsystem GMbH, Wetzlar, Germany). Caco-2 and HepG2 cells with a degree of confluence of approximately 80% were subcultured to maintain their undifferentiated character and therefore the rapid growth of the cells. For this purpose cells were gently rinsed with PBS and subsequently detached from the flask with trypsin-EDTA 0.05% and seeded in a new T25 flask (ratio 1:5). 

### 4.3. Exposure of Cells to Cereulide

The 1 mg·mL^−1^ solution of CER (Chiralix, Nijmegen, The Netherlands) was diluted to working solutions (ranging from 0.05 to 10 nM) in cell culture medium to obtain the ‘exposure’ medium. Chosen concentrations are lower or correspond to previously published minimal doses of CER needed for a measurable response in bioassays and concentrations found in foods placed on the market. These concentrations are also lower than concentrations reported in body fluids of patients diagnosed with severe form of CER intoxication, such as gastric fluid (4 ng·mL^−1^), blood serum (4 ng·mL^−1^), and urine (8 ng·mL^−1^) and stool samples (160–800 ng/g) [52].

The cells were subjected to CER treatment at 80% confluence. The growth medium was replaced by CER containing exposure medium. During the 10 days exposure period, cells were allowed to grow to 80% confluence before splitting. First, the non-exposed cells were counted with a Bürker counting chamber according to the Trypan blue staining method and the number of cells·mL^−1^ of non-exposed cells was obtained. Afterwards, the cells exposed to different CER concentrations were counted and seeded at a cell density equal to that of the non-exposed cells. In this way, after each splitting, cells were seeded equally to obtain the same number of cells for each condition which allowed comparison of the different concentrations. 

### 4.4. Cell Viability and Protein Content Assays

Two widely established assays, MTT ((3-(4.5-dimethylthiazol-2-yl)-2.5-diphenyltetrazolium bromide) and SRB (sulforhodamine B), were used for characterization of the cell viability and cellular protein content after Caco-2 and HepG2 cells were exposed to CER [55,56]. Caco-2 and HepG2 were seeded to each well of the 96-well plates at a density of respectively, 20,000 cells and 30,000 cells per well. Subsequently, the cells continued to grow in exposure medium for 24 h at 37 °C in an incubator with a humidified atmosphere of 5% CO_2_. Mitochondrial activity of the exposed cells was measured after 24 h with the MTT assay. Briefly, 20 µL of MTT dissolved in PBS at 5 mg·mL^−1^ was added to all wells of the assay, and plates were incubated at 37 °C for 2 h to convert MTT to formazan. After incubation, medium was discarded and the purple formazan crystals were solubilized in dimethylsulfoxide (DMSO). Absorbance was recorded at the wavelength of 570 nm by SpectraMax M2 multimode plate reader (Molecular Devices, Sunnyvale, CA, USA). In parallel, the SRB assay was used for cellular protein content measurement. The cell monolayers were fixed with 50 µL of 50% trichloroacetate (TCA) in milliQ-water and stained for 1 h. After washing, cells were stained with 50 µL of SRB solution (0.4% in 1% glacial acetic acid). After 30 min, the plate was rinsed with 1% glacial acetic acid in MilliQ^®^-water and dried for 30 min. The excess was removed by washing repeatedly with 1% glacial acetic acid and the protein-bound dye was dissolved in 10 mM Tris buffer. Absorbance was determined at 490 nm with SpectraMax M2 multimode plate reader. For each cell line and CER concentration, twelve repeats of different cell preparations were made. Both assays were used for the preliminary determination of the sub-emetic concentrations after 1 and 3 days, and as additional tests after a prolonged exposure of 10 days with low doses of CER. 

### 4.5. Seahorse Extracellular Flux Analysis of Mitochondrial Respiration

Mitochondrial respiration in Caco-2 and HepG2 cells was characterized as an indicator of cellular metabolism and fitness in response to the exposure to CER by extracellular flux analysis using Agilent Seahorse XF24 Analyzer (Agilent Seahorse Bioscience, Santa Clara, CA, USA). For this purpose, Agilent Seahorse XF Cell Mito Stress Test was applied to both undifferentiated Caco-2 cells and HepG2 and oxygen consumption rate (OCR) was measured in function of time and added respiration modulators. In short, one day prior to the assay, cells were harvested from the T25 plates in which the cells were exposed to CER. The cells were seeded at 40,000 cells per well (optimized cells density is crucial in the assay [57]) in a Seahorse 24-well XF Cell Culture microplate in 250 µL of the exposure medium and were allowed to adhere for 24 h in a 37 °C humidified incubator with 5% CO_2_. In addition, the Seahorse XF Sensor Cartridge was hydrated the day before running the XF Assay by filling each well of the XF Utility Plate with 1 mL of Seahorse XF Calibrant Solution. The hydrated cartridge was kept in a non-CO_2_ 37 °C incubator for 24 h to remove CO_2_ from the media that would otherwise interfere with measurements that are pH sensitive. On the day of analysis, unbuffered XF Assay Media was used for extracellular flux measurements. For this reason, the cells were washed three times with non-buffered DMEM supplemented with 10 mM glucose, 2 mM sodium pyruvate and 2 mM glutamine (adjusted to pH 7.4) and then maintained in 450 µL·well^−1^ of XF assay media at 37 °C in a non-CO_2_ incubator for 1 h to allow pre-equilibrate with the XF Assay Medium. Mitochondrial function of the cells was analyzed by sequential injections of modulators (with shown final concentration in the wells): oligomycin (1 µM) was used to block ATP synthase, carbonyl-cyanide-4-(trifluoromethoxy) phenyhydrazone (FCCP, 0.25 µM) was used to make the inner mitochondrial membrane permeable for protons and allow maximum electron flux through the electron transport chain, and a mix of rotenone (0.5 µM) and antimycin A (0.5 µM) were used together to inhibit complexes I and III, respectively. These compounds were suspended in pre-warmed XF Assay Medium and loaded into the designated injection ports of the hydrated sensor cartridge corresponding to the order of injection.

Subsequently, the loaded XF Sensor Cartridge with the XF Utility Plate was placed into the XF24 Analyzer and calibrated. After calibration, the XF Utility Plate with the calibration fluid was replaced with the plate containing cells. Each measurement cycle consisted of 3 min of mixing, 2 min of waiting, and 3 min of OCR measurements. First, three basal OCR measurements were performed before the addition of modulators, followed by the sequential addition of oligomycin, FCCP, and rotenone/antimycin A. Measurement cycles were performed after each addition of given compounds. Through use of mitochondrial inhibitors six mitochondrial respiration parameters were determined: basal, ATP production-linked, maximal, proton leak-linked OCR, spare respiratory capacity and non-mitochondrial respiration.

### 4.6. Data Analysis

Statistical analyses were performed using the GraphPad Prism V6.01 software package (GraphPad Software, Inc., San Diego, CA, USA). Comparisons between the control and CER treatments were done by performing one-way analysis of variance (ANOVA) tests followed by post hoc analysis with Dunnett’s multiple-comparison test. Differences were considered significant at *p* < 0.05. Data are presented as mean ± SD.
